# High-throughput variant libraries and machine learning enable robust target-site selection for toehold-switch design in Detectrons

**DOI:** 10.64898/2026.10.09.758053

**Published:** 2026-10-12

**Authors:** Jihoon Han, Seth L Shipman

## Abstract

Programmable RNA sensors enable sequence-specific detection of RNA inputs for diagnostics, genetic circuit control, and multiplexed molecular readouts. Although readily programmable, their performance varies substantially with the target site selected within a transcript, making target-site selection a major challenge. Here, we report DeSTAR (Detectron Sensor TARget-site ranker), a machine-learning-assisted target-site ranking model for programmable RNA sensors. To train this model, we generated transcript-wide, single-nucleotide-resolution tiling sensor libraries across six diverse RNA targets, enabling generalization to be tested across transcripts rather than within a single RNA. In leave-one-transcript-out evaluation, DeSTAR's top-ranked sites were enriched for high-performing sensors in held-out transcripts, driven predominantly by interpretable sequence grammar in the sensor stem rather than transcript-specific patterns. Prospectively, on an unseen SARS-CoV-2 transcript, the model's top-ranked sites yielded working sensors roughly five-fold more often than random selection. These results support DeSTAR as a generalizable strategy for target-site selection in RNA sensors across transcripts.

